# Financial Status of Model, Target, and Observer Modulates Mate Choice Copying and the Mediating Effect of Personality

**DOI:** 10.3390/bs15101324

**Published:** 2025-09-26

**Authors:** Guomei Zhou, Shaxiao Ma, Di Wu

**Affiliations:** 1 Department of Psychology, Sun Yat-sen University, Guangzhou 510006, China; 2 Guangzhou Nansha Qihui Special School, Guangzhou 511400, China; mashx3@mail3.sysu.edu.cn

**Keywords:** mate choice copying, financial status, facial attractiveness, mediating effect, personality, social status, intelligence, social learning

## Abstract

This study investigates the phenomenon of mate choice copying, wherein an individual (the observer) utilizes the assessments of peers of the same sex (the models) regarding a potential partner of the opposite sex (the target), thereby emulating the mate selection preferences of those peers. We examine how the financial status of female models, male targets, and female observers influences mate choice copying, along with the underlying social learning mechanisms. The findings reveal that mate choice copying occurs in the presence of high-financial-status models but is absent when models have low financial status. Mate choice copying, regulated by the financial status of the models, only manifests with targets of low financial status; it does not occur when the target’s financial status is high. Observers with low financial status engage in mate choice copying, irrespective of the models’ financial status. In contrast, observers with high financial status are unaffected by models with a low financial status and only exhibit mate choice copying if the models possess high financial status. Moreover, the study indicates that mediators such as personality traits exert disparate influences on the mate choice copying of observers from different economic backgrounds, suggesting the operation of distinct social learning mechanisms.

## 1. Introduction

### 1.1. General Background

Mate selection is a multifaceted and adaptable process, influenced by a blend of innate predispositions and learned behaviors, shaped by specific circumstances, and susceptible to a variety of social and non-social influences (Cummings & Ramsey, 2015). The inherent uncertainties and costs of choosing a partner—including expenditures of energy and time, opportunity costs, and unforeseen risks—lead individuals to integrate past experiences with advice from family and friends when evaluating prospective mates. Furthermore, individuals often observe and consider the choices made by others of their gender when assessing a potential partner’s suitability for marriage, a phenomenon known as mate choice copying (for reviews, see Anderson & Surbey, 2020; Gouda-Vossos et al., 2018).

**Figure 1:**
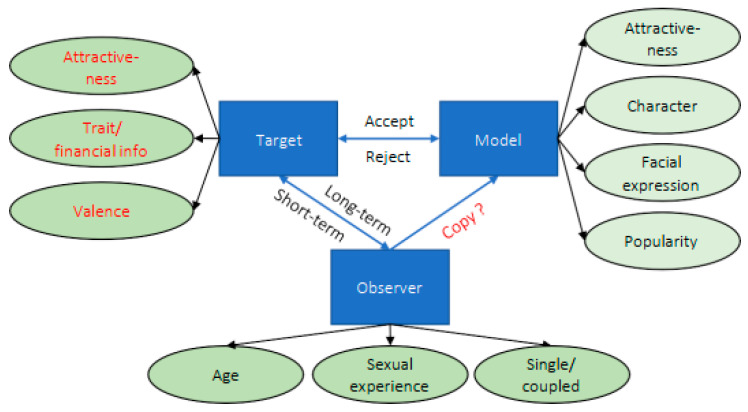
TOM of mate choice copying (adopted from Liu et al., 2021).

For consistent terminology throughout this study, we adopt the definitions outlined by Liu et al. (2021): the *observer* is the female participant making the selection, the *target* refers to the member of the opposite sex being evaluated, and the *model* is the female individual associated with the target. While mate selection is conceptualized as a dyadic interaction between observer and target, mate choice copying involves a triadic interaction among the model, observer, and target, which inherently increases the complexity of influencing factors. Liu et al. (2021) developed the Target-Observer-Model (TOM) framework to explain the dynamics influencing the mate choice copying (Figure 1).

Existing research has primarily focused on factors related to the model, the observer, and the target. However, there is a notable gap in research investigating the impact of financial status on mate choice copying behaviors. While Liu et al. (2021) manipulated target economic information, the broader influence of financial status across observers, models, and targets remains largely unexplored. Numerous studies confirm financial status as a critical consideration in mate selection, particularly for women (Gouda-Vossos et al., 2019; Lu et al., 2015). Despite this, fundamental questions persist regarding whether and how observers of varying economic backgrounds rely on models with diverse financial statuses in their mate selection processes.

### 1.2. Mate Choice Copying

Theoretically, mate choice copying stems from social learning, where individuals glean social information about potential mates by observing the preferences of their same-sex peers. This process provides insights into prospective mates that might not be directly discernible through observation alone, encompassing attributes such as personality traits, financial status, social ranking, and intelligence. This social learning approach influences individual judgments on mate selection, circumventing the need for potentially costly asocial learning—i.e., learning that occurs independently of social interactions (for a broader view, see Kendal et al., 2018). The various factors influencing mate choice copying, as depicted in Figure 1, further support this social learning theory (see Liu et al., 2021, for details). Below, we introduce aspects relevant to the present study.

From a target perspective, research indicates that attractive targets tend to produce stronger mate choice copying effects (Uller & Johansson, 2003). Interestingly, the researchers suggested that participants might perceive it as normal for highly facially attractive men to be partnered, thus failing to extract additional reference information and consequently not exhibiting mate choice copying. Conversely, men with less attractive faces who are partnered might possess unobservable advantages, such as personality or financial status, making mate choice copying more likely to occur. When observers are provided with direct personal information about the target (including personality traits and financial status), mate choice copying tends to disappear (Liu et al., 2021). This is because the female observer has obtained key information about the potential mate and no longer needs to infer it through potentially unreliable social cues.

From an observer’s perspective, many animal studies (e.g., White & Galef, 2000) and human studies (e.g., Bowers et al., 2012; Hill & Buss, 2008; Place et al., 2010) have found that female observers consistently exhibit more stable mate choice copying than male observers. This difference may stem from distinct focuses in mate evaluation (Feingold, 1992). Men often prioritize physical attractiveness and reproductive ability, information that is relatively easy to obtain through direct observation, thereby reducing their reliance on mate choice copying (Zhuang et al., 2012). However, according to parental investment theory (Trivers, 1972), women bear significant risks in childbirth and raising offspring, leading them to prioritize a partner’s willingness to invest resources in offspring. Therefore, women often evaluate potential mates based on personality, social status, financial status, and intelligence (Buss, 1989). Since these traits are not easily discernible through appearance alone, the choices of other women become a valuable source of information (Place et al., 2010). Rodeheffer et al. (2016) provided direct evidence for this social learning inference, finding that female participants’ ratings of male targets across nine dimensions (e.g., intelligent, trustworthy, wealthy) incompletely mediated mate choice copying.

From the perspective of models, the facial attractiveness of female models is believed to reveal qualities of male targets and influence female observers’ mate choice copying (Wang et al., 2021). Male targets paired with high- (vs. low-) facial-attractiveness female models are rated as having higher facial attractiveness (Little et al., 2011). Similarly, the personality (Chu, 2012) and popularity (Little et al., 2015) of female models may also signal qualities of male targets, thereby affecting mate choice copying in female observers. Researchers explain these phenomena using the “desirability heuristic evaluation” theory. When women judge whether a man meets their spousal requirements, they may use other women’s evaluation criteria as a reference (Hill & Buss, 2008). Women with attractive faces and good personalities are often highly selective in their assessments of men, desiring partners with higher social status, intelligence, independence, and confidence (Buss, 1989; Hill & Buss, 2008). Consequently, men selected by high-quality women are more likely to possess desirable personality traits, better economic conditions, higher social status, and a willingness to raise children. Therefore, when such a female serves as a model, observers are more likely to engage in mate choice copying.

## 2. The Present Study

Despite the critical importance of financial status in female mate selection, research has not thoroughly explored its influence on mate choice copying across observers, models, and targets (with the exception of Liu et al., 2021, who manipulated target financial status). Moreover, women’s mate preferences are not static. Under economic pressure, women tend to prefer men with more resources (Tian et al., 2019), while women with high (vs. low) financial status may prioritize qualities representing “good fathers” such as thoughtfulness, patience, loyalty, and caring (Lu et al., 2015). This phenomenon can be explained by the “structurally powerless” hypothesis (Buss & Barnes, 1986; Eagly & Wood, 1999), which posits that women often seek partners with higher socioeconomic status because power and resources are typically controlled by men. Thus, to gain power and resources, women might choose powerful, financially capable men. According to this hypothesis, economically secure women might have lower economic requirements for their spouses as they do not need to acquire power and resources through a partner.

However, some studies present contradictory findings, indicating that women with higher financial status actually have higher financial demands for their spouses. For example, Buss (1989) found that women with high (vs. average) financial status prefer future partners with a higher social and financial status. Wiederman and Allgeier (1992) similarly found that schoolgirls with higher expectations for their future salaries also had higher requirements for the economic prospects of their future spouses. The “desirability heuristic evaluation” theory (Hill & Buss, 2008) can explain these conflicting results.

The present study addresses these gaps by using male faces rated as low in attractiveness as targets, female participants as observers, and female silhouettes as models. We manipulated the relationship between the model and the target, describing the model as either the target’s current girlfriend (as in Rodeheffer et al., 2016) or a high school classmate. This allowed us to investigate mate choice copying and its underlying social learning mechanisms. Experiment 1 manipulated the financial status of the model and asked participants to rate the attractiveness and personality of the target. Experiment 2 manipulated the financial status of both the model and the target, asking participants to rate the attractiveness and personality of the target. Experiment 3 manipulated the financial status of the model and selected participants with different financial statuses, asking them to rate the target’s attractiveness, personality, financial status, personality quality, and intelligence.

This study seeks to answer the following questions and test the accompanying hypotheses:

Question 1: Does the model’s financial status affect mate choice copying in the same way as the model’s facial attractiveness, personality, and popularity?

**Hypothesis** **1.** *We predict an interaction between the relationship and the financial status of models. Specifically, the financial status of models will moderate the mate-choice-copying effect. With high-financial-status models, mate choice copying will occur (i.e., targets paired with a girlfriend model will be rated as more facially attractive than those paired with a classmate model). Conversely, with low-financial-status models, we anticipate no mate choice copying (i.e., no difference in facial attractiveness ratings between girlfriend and classmate conditions).*

Question 2: Previous studies have shown that the mate-choice-copying effect disappears when information about the target’s personality and financial status is available. Does the financial status of the target modulate the impact of the model’s financial status on mate choice copying?

**Hypothesis** **2.** *We hypothesize an interaction between the relationship and the financial statuses of both the model and the target. The model’s financial status is expected to modulate mate choice copying only when targets are of low financial status; mate choice copying should not occur when targets are of high financial status.*

Question 3: Women under economic pressure prefer men with more resources, while women with high socioeconomic status sometimes prioritize “good father” qualities (structurally powerless hypothesis) and other times exhibit higher financial demands for spouses (desirability heuristic evaluation theory). Are there differences among female participants of different financial statuses in whether and why mate choice copying occurs? Does an observer’s financial status moderate the effect of the model’s financial status on mate choice copying?

**Hypothesis** **3.** *We expect an interaction between the relationship and the financial statuses of both model and observer. Specifically, low-financial-status observers will exhibit mate choice copying regardless of whether the model’s financial status. In contrast, high-financial-status observers will be less susceptible to the influence of low-financial-status models, engaging in mating choice copying only when the model is also of high financial status.*

**Hypothesis** **4.1** (Based on the “structurally powerless” hypothesis). *When mate choice copying occurs, low-financial-status observers’ inferences about the target’s economic and social status will increase accordingly, mediating the mate choice copying effect. For high-financial-status observers, their personality ratings of targets will mediate their mate choice copying.*

**Hypothesis** **4.2** (Based on the “desirability heuristic evaluation” hypothesis). *Observers with high financial status will infer the target’s economic and social status accordingly, mediating their mate choice copying.*

The work described has been carried out in accordance with The Code of Ethics of the World Medical Association (Declaration of Helsinki) for experiments involving humans. The experiment was approved by the Institutional Review Board of our department.

## 3. Experiment 1: Model’s Financial Status Affects Mate Choice Copying

### 3.1. Method

#### 3.1.1. Participants

We calculated the optimal sample size of participants in Experiment 1 in accordance with Westfall’s (2016) PANGEA (Power ANalysis for GEneral ANOVA) designs. The estimated sample size for a within-subjects design was 20, with replicates of 24 (24 faces to be evaluated for each participant × relationship × model financial status), from which a significant interaction between relationship and model financial status can be obtained (*d* = 0.45, power = 0.9). Thirty-seven single, heterosexual Chinese female participants were recruited through WeChat and other social media apps (heterosexual only because sexual orientation affects mate choice copying, see Scofield et al., 2020). All participants participated voluntarily, signed an informed consent form before the experiment, and subsequently received 5 CNY for payment. Of the participants, one participant dropped out of the experiment midway, leaving a total of 36 valid participants (*M*_age_ = 20.8, *SD*_age_ = 2.28).

#### 3.1.2. Materials

##### Headshots

We selected 110 original adult male headshots with neutral expressions from the Internet. The images was processed to ensure that grayscale was consistent and that only the face and a small amount of hair were retained. Twenty-six female participants (*M*_age_ = 19.7, *SD*_age_ = 1.51) were recruited to pre-test the attractiveness of these faces. The average attractiveness of the 110 male faces was 3.57 (on a 9-point scale with 1 = very unattractive, 9 = very attractive), distributed between 1.92 and 6.38, with a standard deviation of 0.93.

Ninety-six headshots of men were selected and divided into four groups of 24. There were no significant differences among the four groups in terms of pre-test facial attractiveness ratings (first group, *M* = 3.32, *SD* = 0.73; second group, *M* = 3.32, *SD* = 0.73; third group, *M* = 3.33, *SD* = 0.72; fourth group, *M* = 3.33, *SD* = 0.71), *F*(3,75) = 0.276, *p* = 0.842.

##### College Students’ Subjective Social Status Scale

The questionnaire is taken directly from Cheng et al. (2015). It had seven questions, related to academic performance, family conditions, popularity status, social practice ability, talent level, relationship status (single or in love) satisfaction, and looks (1 = lowest status, 10 = highest status in your university on a ladder picture). Cronbach α for this questionnaire was 0.78 (Cheng et al., 2015).

#### 3.1.3. Design

This experiment adopted a 2 (relationship: girlfriend, classmate) × 2 (model financial status: 5000 CNY, 20,000 CNY) within-subjects design. The subjective social status of the participants was measured using the “College Students’ Subjective Social Status Scale”.

This relationship refers to the design of Rodeheffer et al. (2016). The condition of “current girlfriend” means that the man is currently in a relationship with the woman, whereas the condition of “high school classmate” means they are just classmates.

In the experiment, the model is described as having either a high (20,000 CNY) or a low (5000 CNY) monthly income. These values were chosen based on the “2019 Job Search Trend Report for Fresh Graduates” (BOSS Direct Recruitment, 2019). The report pointed out that the average salary of new undergraduate graduates was 5909 yuan. With reference to this average salary, a monthly income of 5000 CNY represents a low financial status, and a monthly income three times the average salary, 17,727 CNY, a high salary. To avoid complicated numbers that might confuse the participants, we set a monthly income of 20,000 CNY as representing a high financial status.

The dependent variable refers to Rodeheffer et al.’s (2016) design and is the mean of the following three 9-point Likert scale ratings: 1. “How desirable is this man as a romantic partner?” (1 = *very undesirable*; 9 = *very desirable*); 2. “How attractive is this man?” (1 = *very unattractive*, 9 = *very attractive*); 3. “How romantically appealing is this man?” (1 = *very unappealing*, 9 = *very appealing*). A fourth question was added, “How likely is this man to have a good personality?” (1 = *very unlikely*, 9 = *very likely*) to measure the participant’s inferred score for the individual’s personality.

#### 3.1.4. Procedure

E-Prime 2.0 was used for the experiments. First, participants were asked to rate the 96 male headshots. These were divided into four groups, one for each experimental condition, and the matching order was balanced across the participants. The experimental conditions were as follows. The 24 headshots in each set were presented randomly within that set. In each trial, two images appeared in the center of the screen: a male face and a female silhouette. Text above the female silhouette image described the woman as either the man’s current girlfriend or a high school classmate, while the woman’s income appeared at the bottom. Questions to be scored and the corresponding 9-point scales appeared below the faces 3 s after the information was presented. After completing the rating, participants automatically proceeded to the next rating session. There were four rating questions. The order in which the first three questions for attractiveness were presented was counterbalanced across participants. The question for personality was always presented lastly. Picture materials and text information were presented on the screen until the participants had completed all the questions.

Next, participants completed the College Students’ Subjective Social Status Scale. The participants were asked to imagine that the ten ladder beams in the ladder picture represented their current status in the university they were currently studying at and to use the mouse to click on the beam of the corresponding status. Once this had been done, the next question automatically appeared. Seven questions of the College Students’ Subjective Social Status Scale were presented randomly.

### 3.2. Results and Discussion

#### 3.2.1. Facial Attractiveness Rating

The mean of the first three ratings was used as the dependent variable for facial attractiveness. We conducted a 2 (relationship: girlfriend, classmate) × 2 (model financial status: 5000 CNY, 20,000 CNY) repeated measures ANOVA on facial attractiveness scores.

The main effect of the relationship was significant, *F*(1,35) = 12.39, *p* = 0.001, $\eta_{p}^{2}\;$ = 0.261, with a higher rating for girlfriend (*M* = 4.25) than for classmate (*M* = 4.07). In other words, mate choice copying occurred. The main effect of the model’s financial status was significant, *F*(1,35) = 12.21, *p* = 0.001, $\eta_{p}^{2}$ = 0.259, with a higher rating under the 20,000 CNY condition (*M* = 4.39) than the 5000 CNY condition (*M* = 3.94).

**Figure 2:**
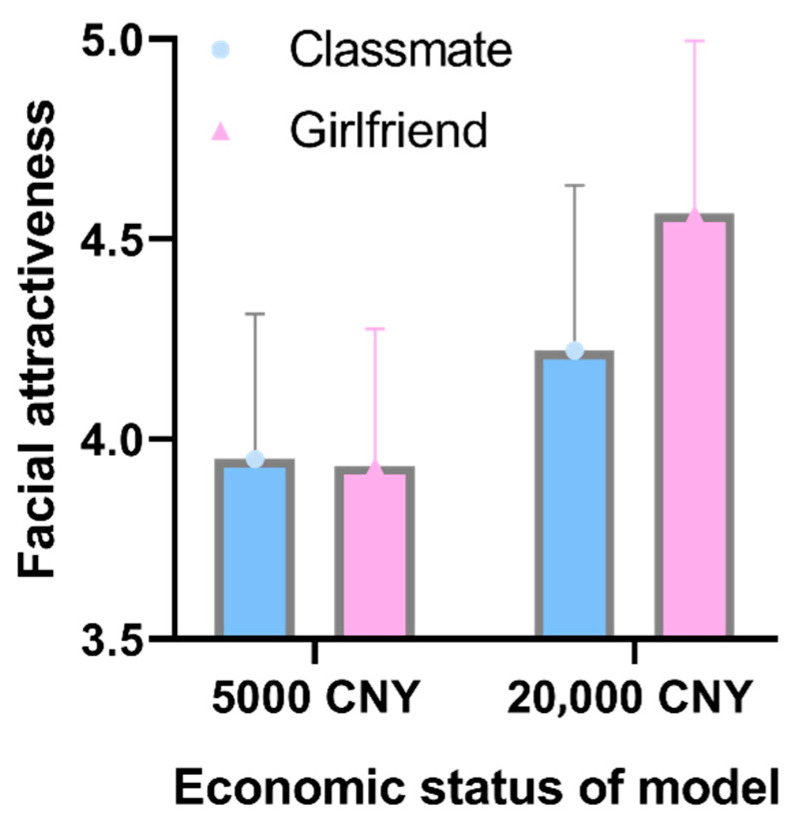
The impact of the financial status of model on the mate-choice-copying effect in Experiment 1. Error bars represent the 95 % confidence interval.

The interaction effect of relationship × model financial status was significant, *F*(1,35) = 15.81, *p* < 0.001, $\eta_{p}^{2}$ = 0.311. The results of the simple effects analysis (see Figure 2) show that mate choice copying occurred only when the model earned 20,000 CNY each month (the rating in the girlfriend condition (*M* = 4.56) is significantly higher than that in the classmate condition (*M* = 4.22)); *p* < 0.001. However, when the model earned 5000 CNY each month, there was no mate choice copying; the rating in the girlfriend condition (*M* = 3.93) was not significantly different from that in the classmate condition (*M* = 3.95), *p* = 0.74. This result verifies Hypothesis 1 and is consistent with previous results on model facial attractiveness (Little et al., 2011), model personality (Chu, 2012), and model popularity (Little et al., 2015). The financial status of the models is a key factor affecting mate choice copying.

#### 3.2.2. Mediating Effect of Perceived Personality

The value of item 4 (How likely is this man to have a good personality?) was used as the mediating variable for the personality scores. Because this study adopted a within-subjects design, the MEMORE plug-in for SPSS 30.0 (Montoya & Hayes, 2017) was used to calculate the mediating effect of personality on relationships and facial attractiveness under low and high financial status.

The results (see Table 1) show that personality has a significant mediating effect on the impact of relationship on facial attractiveness only for high-financial-status models, which is an incomplete mediator. When a model with high financial status is paired with the target, female participants may speculate that the target has a better personality and will thus be chosen by the model. Therefore, the female participants copied the model’s choices during mate selection. This is also consistent with the results of Rodeheffer et al. (2016), who found that personality mediates mate choice copying.

#### 3.2.3. Subjective Social Status

SPSS was used to conduct a reliability test on the “College Students’ Subjective Social Status Scale.” The scale has seven items, and Cronbach’s α = 0.754, meaning the reliability of the scale in Study 1 was good.

The mean subjective social status of the participants was 6.08, standard deviation 1.15, median 6.21, minimum value 3.57, and maximum value 8.14. The scale scores ranged from 1 (lowest status) to 10 (highest status).

Considering that mate copying occurs only when the model earns 20,000 CNY, we only analyzed the mate-choice-copying effect under this condition. The Pearson correlation between the mate-choice-copying effect in this condition and the participant’s subjective social status score was not significant, *r* = −0.135, *p* = 0.432. This may be because, as the results above show, the subjective socioeconomic status perceived by most college students was at the middle level. Moreover, with only 36 participants, the sample sizes of participants with high and low subjective socioeconomic statuses were small. Therefore, in Experiment 2, we used experimental methods to prime participants’ subjective sense of social status with text materials (Lu et al., 2015) and explored their impact on mate choice copying. Experiment 2 also manipulated the targets’ financial status.

## 4. Experiment 2: Financial Status of the Target Modulates the Impact of the Financial Status of the Model on Mate Choice Copying

During the collection of data for Experiment 2, the school laboratory was closed owing to the nationwide COVID-19 epidemic. The Theus psychological experimental system was therefore used to collect the data for Experiment 2. This system is an e-Prime-based online data collection platform for psychological experiments that was opened free of charge during the pandemic by Beijing Hunyuan Times Technology Co., Ltd. (Beijing, China) and the National Experimental Teaching Demonstration Center of Psychology of South China Normal University. The experimenter uploaded the designed E-Prime experiment file to the server to obtain the experiment code. The participants first installed the plug-in and then logged in remotely to enter the experimental system, using the experiment code provided by the experimenter, and completed the E-Prime experiment. The results were automatically uploaded to the server and the host viewed and downloaded the experimental results. The main tester received approximately 20–30 min of remote training before using the device for the first time.

### 4.1. Method

#### 4.1.1. Participants

According to PANGEA (Westfall, 2016) we calculated the optimal sample size of participants for Experiment 2. The estimated sample size of each group of participants in the mixed design was six with replicates of 13 (each participant × relationship × model financial status × target financial status had 13 faces to be evaluated), from which a significant interaction between relationship, model financial status, target financial status, and observer financial status (*d* = 0.45, power = 0.928) can be derived. Ninety single heterosexual female participants were recruited using social media apps. Of these, 42 exited the experiment at some point because of a failure to install and run the plug-in, leaving 48 participants (*M_age_* = 21.8, *SD_age_* = 2.01) who successfully completed the experiment. Of these, 32 completed the experiment through the online E-Prime experimental system, and 16 using E-Prime 2.0 software on their own computer. All participants voluntarily participated and received 10 CNY for payment after the experiment. To eliminate any confounding of the experimental results by other irrelevant variables, we asked the participants who participated in Experiment 2 remotely to be alone in a quiet room where they would not be disturbed, to set their mobile phones to “do not disturb mode,” to adjust the computer screen brightness to 100 and resolution to 1024 × 786 pixels, and to close computer software and browser web pages not related to the experiment.

#### 4.1.2. Design

This experiment used a 2 (relationship: girlfriend, classmate) × 2 (model financial status: 5000 CNY, 20,000 CNY) × 2 (target financial status: 5000 CNY, 20,000 CNY) × 2 (observer financial status: low, high) mixed design. The relationship, the model financial status, and the target financial status were within-subject variables, and the settings for each condition were consistent with Experiment 1. Observer financial status was the between-subject variable. The “College Student Subjective Social Status Scale” was used to determine whether manipulation of the observer’s financial status was successful. The dependent variables and mediating variables were identical with Experiment 1.

#### 4.1.3. Materials

We randomly selected 104 male headshots as the experimental materials from among the 110 headshots previously tested. The participants were divided into eight groups with 13 headshots in each group. There were no significant differences between the eight groups in terms of male facial attractiveness ratings, *F*(7,175) = 0.276, *p* = 0.842 (first group, *M* = 3.46, *SD* = 0.89; second group, *M* = 3.45, SD = 0.91; third group, *M* = 3.44, *SD* = 0.89; fourth group, *M* = 3.43, *SD* = 0.85; fifth group, *M* = 3.43, *SD* = 0.78; sixth group, *M* = 3.45, *SD* = 0.81; seventh group, *M* = 3.47, *SD* = 0.81; eighth group, *M* = 3.48, *SD* = 0.83).

#### 4.1.4. Procedure

E-Prime 2.0 was used for the experiments. The specific experimental procedure was as follows. Participants were randomly assigned to two groups.

Participants in the high-financial-status group of observers read the following instructions: “Compared with the past, people in this era are more in control of their lives, have more abundant resources, and women can easily make ends meet. In the next three minutes, please imagine that you have enough financial resources and abundant material resources, and how you will use your money in various types of consumption such as houses, cars, furniture, etc.” (Lu et al., 2015).

Participants in the low-financial-status group read the following instructions: “Compared with the past, people in this era are less able to control their lives, have more limited resources, and it is difficult for women to make ends meet. In the next three minutes, please imagine that you do not have sufficient financial or material resources, and consider how you will pay off debts and purchase necessities in various types of consumption, such as hospitals, bills, rent, and food (Lu et al., 2015).

Next, the participants entered the face-rating section, where the instructions were the same as in Experiment 1. They rated 104 faces in eight groups. Each set of faces was matched to one experimental condition and the order of matching was counterbalanced across participants, with the 13 faces presented randomly within each block. The procedure for each trial was the same as in Experiment 1, except that the man’s monthly income was displayed directly below the male headshot.

The participants then completed the “College Student Subjective Social Status Scale” to test whether the manipulation of the observer’s financial status was successful. This procedure was consistent with that used in Experiment 1.

### 4.2. Results and Discussion

#### 4.2.1. Manipulation Check on Observer’s Financial Status

First, SPSS was used to conduct a reliability test on the “College Student Subjective Social Status Scale,” giving a Cronbach α = 0.739, indicating good reliability for Experiment 2.

An independent samples *t*-test was used to test whether the manipulation of the observer’s financial status was effective. The results showed no significant difference between the subjective social status scores of the low-financial-status group (*M* = 5.81, *SD* = 0.90) and those of the high-financial-status group (*M* = 5.78, *SD* = 0.95), *t*(46) = 0.13, *p* = 0.90. This result suggests that the experiment may not have successfully manipulated the observer’s financial status. Therefore, the variable of the observer’s financial status was not considered in subsequent analyses.

#### 4.2.2. Facial Attractiveness Rating

A 2 (relationship: current girlfriend, high school classmate) × 2 (model financial status: 5000 CNY, 20,000 CNY) × 2 (target financial status: 5000 CNY, 20,000 CNY) repeated-measures ANOVA was performed on the facial attractiveness ratings in Experiment 2, and all variables were within subject.

The results are summarized in Table 2. The significant main effect of the target’s financial status indicated that the facial attractiveness of the target with a high financial status (*M* = 4.61) was significantly higher than that of the target with a low financial status (*M* = 3.66). The trend in the main effect of the model’s financial status showed that the facial attractiveness of the target in the high-financial-status model condition (*M* = 4.09) was lower than that in the low-financial-status model condition (*M* = 4.17).

Importantly, the relationship × model financial status × target financial status interaction was significant. The results of the simple effects analysis are presented in Table 3. Mate choice copying occurs only when high-economic-level models are paired with low-economic-level targets. In the other cases, mate choice copying did not occur. In other words, the influence of the model’s financial status on mate choice copying found in Experiment 1 only occurred when the target’s financial status was low, thus verifying Hypothesis 2.

#### 4.2.3. Mediating Effect of Perceived Personality

Consistent with the mediation analysis in Experiment 1, the mediating effect of personality on the influence of relationships on facial attractiveness was analyzed for four situations consisting of low/high-financial-status models and low/high-financial-status targets (see Table 4). Under the conditions of mate choice copying in this experiment (i.e., a high-financial-status model paired with a low-financial-status target), the mediating effect of perceived personality was significant, as was the total effect. This is consistent with previous work (Rodeheffer et al., 2016) and the results of Experiment 1. The reason mate choice copying occurs under this condition may be because observers speculate that the low-financial-status target is selected by the high-financial-status model because it has a better personality.

## 5. Experiment 3: Observer Financial Status Modulates the Impact of Model Financial Status on Mate Choice Copying

The existing literature points out that factors that may affect women’s mate selection include men’s financial status, social status, and intelligence (Li et al., 2013; Lu et al., 2015; Prokosch et al., 2009). Therefore, this experiment added the measurement of these three mediating factors to the previous experiment.

Experiment 3 was conducted at a time when the COVID-19 epidemic was rebounding. Many cities implemented a refined management strategy and many companies switched to remote working. At this time, it was very difficult to collect participant data on-site; therefore, the Brain Island scientific research platform (naodao.com) was adopted to conduct the online experiments.

### 5.1. Method

#### 5.1.1. Participants

Experiment 3 mainly explored mate choice copying by women of different financial statuses. Therefore, we chose both university students and working women as participants. To prevent participants from worrying about disclosing specific information about their incomes, participants were divided into three levels by income for this experiment (annual pre-tax income of below 25,000 CNY of participants from Naodao, between 120,000 CNY and 240,000 CNY working women, and above 240,000 (20,000 × 12) CNY working women). Because the overall income of the participants provided on the Naodao platform is relatively low, in Experiment 3, only participants with low financial status were recruited through Naodao, and participants with medium and high financial status were mainly company employees.

Again, we calculated the participant sample size for Experiment 3 based on PANGEA (Westfall, 2016). The estimated optimal sample size of each financial status participant group in the mixed design was 10, with 24 replicates (24 faces were evaluated by each participant) to obtain a significant interaction between relationship, model financial status, and the observer’s financial status (*d* = 0.45, power = 0.91). To balance the presentation order of the different faces and questions, we recruited more participants. Finally 91 unmarried heterosexual women (*M*_age_ = 26.54; *SD*_age_ = 4.425) participated in the experiment. Of these, 31 reported an annual pre-tax income less than 25,000 CNY (low); 30 between 120,000 CNY and 240,000 CNY (medium); and 30 greater than 240,000 CNY (high). All participants signed informed consent forms and received 15 CNY after completing the experiment.

#### 5.1.2. Design

Experiment 3 adopted a 2 (relationship: current girlfriend, high school classmate) × 2 (model financial status: 5000 CNY, 20,000 CNY) × 3 (observer financial status: low, medium, high) mixed design. The last variable was the between-subject variable.

Experiment 3 collected participants’ data online. The three questions corresponding to the attractiveness rating were “How satisfactory do you think this man would be as a love partner?” “How attractive do you find this man’s face?” “How likely are you to choose this man as a romantic partner?” The average score of the three questions was used as the participant’s rating for the target’s attractiveness. In addition, Experiment 3 included four questions about the possible mediating factors of the mate-choice-copying effect: “Do you think it is possible that this man has a good financial status?” “Do you think it is possible that this man has a high social status?” “Do you think it is possible that this man has a good personality?” “Do you think it is possible that this man has a high intelligence?” This explored the participant’s assessments of the target’s financial status, social status, personality, and intelligence, respectively. The seven questions were scored on a 9-point Likert scale (1 = very impossible, 9 = very possible).

#### 5.1.3. Procedure

The male headshots and female silhouettes used in Experiment 3 were identical to those used in Experiment 1.

The program for Experiment 3 was written using Psychopy 2020 software, and the procedure was essentially the same as for Experiment 1. The program randomly presented 96 male headshots and asked participants to rate them. The four independent variable combination conditions “current girlfriend, monthly income of 20,000 CNY; current girlfriend, monthly income of 5000 CNY; high school classmate, monthly income of 20,000 CNY; high school classmate, monthly income of 5000 CNY” were randomly combined with headshots, and the trial number for each condition within the participants was balanced (24 times for each combination condition). The procedure for each trial was identical to that used in Experiment 1. The first three questions were presented to each participant in a random order. The order of the three questions was fixed across different trials within each participant. After the first three questions were presented, questions 4–7 were also presented in a random order between participants and in a fixed order within participants. While answering these questions, male headshots, female silhouettes, and text information were presented on the screen. After all seven questions had been answered, the next male headshot was presented until all 96 sets had been presented and the experiment ended.

### 5.2. Results and Discussion

The average score of the first three questions was used as the dependent variable for the participant’s rating of the target’s attractiveness, and the scores of questions 4–7 were used as the dependent variable for the participant’s inference of the target’s financial status, social status, personality, and intelligence. SPSS was used to conduct a 2 (relationship: current girlfriend, high school classmate) × 2 (model financial status: 5000 CNY, 20,000 CNY) × 3 (observer financial status: low, medium, high) mixed-design MANOVA analysis. The MEMORE plug-in in SPSS was used to conduct analyses, with financial status, social status, personality quality, and intelligence as mediating variables.

#### 5.2.1. Facial Attractiveness Rating

The results for facial attractiveness (see Table 5) show a significant main effect of this relationship. The target’s attractiveness under the girlfriend condition (*M* = 3.565) was significantly higher than that under the classmate condition (*M* = 3.258), indicating a mate-choice-copying effect.

The main effect of the model’s financial status was significant. The attractiveness of the target’s face in the high-financial-status model condition (*M* = 3.733) was significantly higher than that in the low-financial-status model condition (*M* = 3.090).

The interaction between the relationship and model financial status was significant. The results of the simple effect test showed that under the condition of models with high financial status, the main effect of the relationship was significant (*M_g_*_irlfriend_ − *M*_classmate_ = 0.419, *p* < 0.001); under the condition of models with low financial status, the main effect of the relationship was also significant (*M_g_*_irlfriend_ − *M*_classmate_ = 0.195, *p* = 0.004). This result is inconsistent with those of Experiment 1, possibly because of the introduction of participants with different financial statuses in Experiment 3. This suggestion is supported by other results derived from this data.

The interaction between relationship and observer’s financial status was significant, *F*(2,89) = 3.240, *p* = 0.044, $\eta_{p}^{2}$ = 0.069, indicating that observers with different financial statuses exhibited different mate-choice-copying effects. The results of the simple effect test showed that mate choice copying did not occur with observers with low financial status (*M_g_*_irlfriend_ − *M*_classmate_ = 0.144, *p* = 0.140) but did for medium-financial-status (*M_g_*_irlfriend_ − *M*_classmate_ = 0.494, *p* < 0.001) and high-financial-status observers (*M_g_*_irlfriend_ − *M*_classmate_ = 0.282, *p* = 0.005).

The interaction between relationship, model’s financial status, and observer’s financial status was significant, *F*(2,89) = 7.168, *p* = 0.001, $\eta_{p}^{2}$ = 0.140, indicating that the observer’s financial status moderated the impact of the model’s financial status on mate choice copying, thus verifying Hypothesis 3. The scores for each condition are listed in Table 6.

Statistical analysis was conducted separately for participants with different financial statuses. The results showed that the interaction between relationship and model’s financial status was not significant for participants with low [*F*(1,30) = 0.899, *p* = 0.351, $\eta_{p}^{2}$ = 0.029] or medium financial status [*F*(1,29) = 0.287, *p* = 0.596, $\eta_{p\;}^{2}$ = 0.010]. However, it was significant for participants with high financial status, *F*(1,29) = 26.497, *p* < 0.001, $\eta_{p\;}^{2}$ = 0.477. Further analysis found that observers with low/medium financial status exhibited mate choice copying or trends regardless of whether the model’s financial status was high or low. However, for observers with high financial status, when the model’s financial status was high, the participants exhibited mate choice copying (*M_g_*_irlfriend_ − *M*_classmate_ = 0.549, *p* < 0.001), but when the financial status of the model was low, they did not (*M_g_*_irlfriend_ − *M*_classmate_ = 0.015, *p* = 0.848).

#### 5.2.2. Rating of Financial Status, Social Status, Personality, and Intelligence

The MANOVA results for financial status, social status, personality quality, and intelligence evaluation (see Table 5) show that, in all dimensions, the interaction between the relationship and the model’s financial status was significant and not moderated by the observer’s financial status. The results of the simple effect test showed that when the target had a current girlfriend with a high financial status (vs. a high school classmate with a high financial status), the participants thought that the target had a higher financial status (*p* < 0.001), higher social status (*p* < 0.001), better personality quality (*p* < 0.001), and higher intelligence (*p* < 0.001). When the target had a current girlfriend with a low financial status (vs. a high school classmate with a low financial status), participants did not exhibit these beliefs (*p* > 0.15), except for personality quality (*p* = 0.017). In other words, regardless of whether the target ‘s girlfriend had a high or low financial status, the participants believed that the target’s personality was better, but the difference in personality inference when the target had a high-financial-status girlfriend (vs. high-financial-status high school classmate) (*M* = 0.598) was significantly higher than the personality inference difference (*M* = 0.176) when the target had a current girlfriend (compared to a high school classmate) with a low financial status.

In the inference of social status and intelligence, the interaction between the relationship and observer’s financial status had a trend in *p* values (*p* = 0.061 for social status, *p* = 0.05 for intelligence). However, the $\eta_{p}^{2}$ values are all greater than 0.06, indicating a medium level, showing that the financial status of the observer at this time had a significant impact on the mate-choice-copying effect on social status and intelligence inference. The results of the simple effect test on social status showed that observers with low financial status only showed a tendency toward mate choice copying (*M_g_*_irlfriend_ − *M*_classmate_ = 0.258, *p* = 0.058); observers with medium financial status showed obvious mate choice copying (*M_g_*_irlfriend_ − *M*_classmate_ = 0.678, *p* < 0.001); and observers in the high-financial-status group also showed mate choice copying (*M_g_*_irlfriend_ − *M*_classmate_ = 0.298, *p* = 0.032). The results of the simple effect test on intelligence showed similar results, with low-financial-status observers showing no mate choice copying (*M_g_*_irlfriend_ − *M*_classmate_ = 0.160, *p* = 0.224); observers with medium financial status showing obvious mate selection replication (*M_g_*_irlfriend_ − *M*_classmate_ = 0.622, *p* < 0.001); and observers with high financial status also showing mate choice copying (*M_g_*_irlfriend_ − *M*_classmate_ = 0.369, *p* = 0.007).

#### 5.2.3. Mediating Effect of Financial Status, Social Status, Personality, and Intelligence

According to Hypothesis 4.1 and Hypothesis 4.2, the impact of the observer’s financial status on mate choice copying is realized through one or more of participants’ financial status, social status, personality, and intelligence, with other target factors as mediating factors. Therefore, we calculated the mediating effects of financial status, social status, personality, and intelligence inference on mate choice copying for different observers’ financial statuses (see Table 7).

Analysis of the mediating effect for observers with different financial statuses showed it was mainly reflected in social status and intelligence, similar to the MANOVA results. Specifically, social status plays a complete mediating effect on the mate choice copying of low- and medium-financial-status observers, but only partially mediates the effect on high-financial-status observers, indicating that high-financial-status observers place less emphasis on social status. The mediating effect of intelligence for observers with low financial status is not significant, but is partial for observers with medium financial status and reaches full mediation for observers with high financial status, indicating that participants with higher financial status are more likely to infer intelligence. Financial status and personality were valued by participants of all financial statuses. Both Hypothesis 4.1 and Hypothesis 4.2 are therefore supported.

We also calculated the mediating effects of financial status, social status, personality, and intelligence on mate choice copying under different financial statuses using the models (see Table 8).

It can be seen from Table 8 that for high-financial-status models, inferences of financial status, social status, personality, and intelligence all have significant incomplete mediation effects. For the low-financial-status model, only personality factors had a significant full mediating effect on participants’ mate choice copying.

## 6. General Discussion and Conclusions

Across three experiments, this study investigated the impact of target, model, and observer’s financial status on mate choice copying and the underlying social learning mechanisms. Our findings reveal four key results, which are discussed in detail below.

### 6.1. Model Financial Status as a Key Factor in Mate Choice Copying

A consistent finding across all three experiments was that the model’s financial status significantly impacts mate choice copying, with copying only occurring when the target was associated with a high-financial-status model. This extends previous research demonstrating the influence of model facial attractiveness (Little et al., 2011), personality (Chu, 2012), and popularity (Little et al., 2015) on mate choice copying. Our results highlight that a model’s financial status is a crucial factor in this phenomenon. Future research should consider manipulating model quality not only through facial attractiveness (Wang et al., 2021) but also through their perceived economic standing.

### 6.2. Target Financial Status Modulates Model’s Influence on Mate Choice Copying

Liu et al. (2021) previously reported that providing any economic information about the target (regardless of whether it was high or low status) eliminated mate choice copying. In contrast, our study found that mate choice copying did occur under specific conditions: when targets had low financial status but were associated with a high-financial-status model. This discrepancy might stem from Liu et al. (2021) only providing personal economic information about the target, without concurrently presenting information about the model’s financial status. In such a scenario, observers, having received direct personal information about the target, would not need to infer information from the model’s choice, thus leading to the absence of mate choice copying.

In our study, however, observers simultaneously received both personal information about the target’s low financial status and social information about the model’s high financial status. This significant contrast likely led participants to prioritize the social information from the high-financial-status model. They may have rationalized the low-financial-status target’s association with a high-financial-status model by inferring that the target possessed other desirable, unobservable qualities, such as a better personality. This inference is supported by our finding of a personality mediation effect. This result aligns with Liu et al.’s (2021) proposition that observers engage in a trade-off between personal and social information when making mate choices.

### 6.3. Observer Financial Status Modulates Model’s Influence on Mate Choice Copying

While Experiments 1 and 2 did not successfully manipulate observer financial status, Experiment 3, by selecting participants with varying annual incomes, revealed that the observer’s financial status modulates the impact of the model’s financial status on mate choice copying. Specifically, low- and medium-financial-status observers exhibited mate choice copying regardless of the model’s financial status. However, high-financial-status observers only engaged in mate choice copying when the model also had a high financial status and did not copy the choices of low-financial-status models.

This differential effect may be attributed to low- and medium-financial-status women potentially having less experience, confidence, or capability in independently acquiring social information about targets, making them more inclined to rely on models for mate selection (Kavaliers et al., 2017). Their behavior could also reflect risk-avoidance strategies given their financial status. Conversely, for high-financial-status observers, guided by the “desirability heuristic evaluation” theory (Hill & Buss, 2008), they may have higher standards for a potential mate than a low-financial-status model might represent. Consequently, high-financial-status observers did not replicate the choices of low-financial-status models. Importantly, the present high- and low-status groups also differed in age; future studies should therefore isolate or statistically control for this potential confound.

### 6.4. Different Social Learning Mechanisms Across Observers of Different Financial Statuses

All three experiments consistently demonstrated a mediating effect of personality inference on mate choice copying, aligning with previous work (Rodeheffer et al., 2016). Furthermore, Experiment 3 provided critical insights into how different financial statuses influence these social learning mechanisms. For observers of varying economic backgrounds, both personality and financial status played a complete mediating role. This suggests that these traits are universally important in the social learning mechanisms of mate choice copying.

However, interesting distinctions emerged for other mediating factors. Social status played a complete mediating role for low- and medium-financial-status observers, but only a partial mediating role for high-financial-status observers, indicating that high-financial-status observers may place less emphasis on social status. Similarly, the mediating effect of intelligence was not significant for low-financial-status observers, a partial mediation for medium-financial-status observers, and a full mediation for high-financial-status observers. This suggests that participants with higher financial status are more likely to infer intelligence in a potential mate. These findings underscore that the mechanisms driving mate choice copying differ among women of varying financial statuses. While our results align with Lu et al.’s (2015) finding that low-financial-status women prioritize resources like social status, they diverge from their finding that high-financial-status women focus more on personality factors. It’s plausible that women with high financial status have elevated standards across multiple dimensions, including financial status, personality, and intelligence. As Buss and Shackelford (2008) observed, “high-quality” women often evaluate potential mates across a broad spectrum of qualities (e.g., good genes, investment ability, parenting ability, and spousal qualities). The precise mechanisms of mate choice copying among participants with different financial statuses warrant further exploration in future research.

### 6.5. Conclusions and Future Directions

In summary, this study makes several significant contributions to the literature on mate choice copying. First, we demonstrate that the financial status of both models and observers influences mate choice copying, enriching our understanding of this phenomenon and extending Liu et al.’s (2021) Target-Observer-Model (TOM) framework. Second, our findings suggest that the mechanisms of mate copying among women of different financial statuses may vary. Future studies should broadly explore the underlying reasons for these differences and how they affect the mate choice copying phenomenon. Finally, this study identifies interactive effects of multiple factors on mate choice copying, further illustrating that it is a complex phenomenon requiring a trade-off among various considerations.

In the world of dating, this research suggests a woman’s decision to follow a friend’s lead isn’t automatic or random. Instead, it’s a calculated choice shaped by the financial status of everyone involved. This research shows that social learning and economic backgrounds work together to create our dating preferences in a complex, non-random way.

Looking forward, the phenomenon of mate choice copying could be formally analyzed using social learning computational models, building upon existing social simulation models of mate selection (Conroy-Beam et al., 2022) and Bayesian models of mate choice copying (Uehara et al., 2005). It is also important to consider that social learning in mate choice copying may not have evolved exclusively for mate selection, but rather as a specialized instance of domain-general social facilitation that developed in other social contexts (see Foreman & Morgan, 2023; Rosenthal & Ryan, 2022; Street et al., 2018). Therefore, future research on mate choice copying should extend beyond the narrow confines of mate selection and embrace cross-disciplinary investigations related to broader social learning principles.

## Figures and Tables

**Table 1:** Mediating effect results of personality in the influence of relationship on facial attractiveness.

	Low Financial Status Model				High Financial Status Model			
Effect	Effect	SE	Statistics	*p*	Effect	SE	Statistics	*p*
Total	−0.018	0.051	*t* = −0.342	0.735	**0.343**	**0.076**	***t* = 4.522**	**<0.001**
Direct effect	−0.044	0.048	*t* = −0.901	0.374	**0.213**	**0.073**	***t* = 2.916**	**0.006**
Indirect effect	0.026	0.023	*Z* = 1.121	0.262	**0.130**	**0.054**	***Z* = 2.418**	**0.016**

Note: Significant results are in bold.

**Table 2:** Statistical results of repeated measures ANOVA in Experiment 2 (*N* = 48).

Effect	*F*	*df*	*p*	$\eta_{p}^{2}$
Relationship (R)	0.047	1.47	0.829	0.001
*Model financial status (M)*	*3.975*	*1.47*	*0.052*	*0.078*
**Target financial status (T)**	**111.917**	**1.47**	**0.000**	**0.704**
**R** × **M**	**8.036**	**1.47**	**0.007**	**0.146**
**R** × **T**	**8.343**	**1.47**	**0.006**	**0.151**
*M* × *T*	*2.966*	*1.47*	*0.092*	*0.059*
**R** × **M** × **T**	**11.386**	**1.47**	**0.001**	**0.195**

Note: Significant results are in bold; trending results are in italics.

**Table 3:** Simple effect analysis of the interaction of relation × model financial status × target financial status (*N* = 48).

Financial Status		Relationship		*F*	*p*
Model	Target	Girlfriend	Classmates		
*Low*	*Low*	*3.68*	*3.78*	*3.56*	*0.065*
**High**	**Low**	**3.72**	**3.45**	**11.51**	**0.001**
Low	High	4.61	4.65	0.68	0.413
*High*	*High*	*4.55*	*4.65*	*2.86*	*0.098*

Note: Significant results are in bold; trending results are in italics.

**Table 4:** Mediating effect results of personality ratings in Experiment 2.

	Low Financial Status Model				High Financial Status Model			
Effect	Effect	SE	Statistics	*p*	Effect	SE	Statistics	*p*
Low financial status target								
Total	*−0.096*	*0.051*	*t = −1.888*	*0.065*	**0.263**	**0.077**	***t* = 3.392**	**0.001**
Direct	−0.056	0.043	*t* = −1.283	0.206	*0.098*	*0.052*	*t = 1.904*	*0.063*
Indirect	−0.040	0.030	*Z* = −1.348	0.178	**0.165**	**0.064**	***Z* = 2.571**	**0.010**
High financial status target								
Total	−0.039	0.047	*t* = −0.826	0.413	*−0.098*	*0.058*	*t = −1.690*	*0.098*
Direct	−0.015	0.042	*t* = −0.346	0.731	−0.025	0.047	*t* = −0.539	0.592
Indirect	−0.024	0.024	*Z* = −1.035	0.301	*−0.072*	*0.039*	*Z = −1.859*	*0.063*

Note: Significant results are in bold; trending results are in italics.

**Table 5:** MANOVA results of in Experiment 3.

		MSE	*F*	*p*	$\eta_{p}^{2}$
Relationship (R)	**Attractiveness**	**8.555**	**29.401**	**0.000**	**0.250**
	**Financial status**	**16.369**	**29.084**	**0.000**	**0.248**
	**Social status**	**15.387**	**27.415**	**0.000**	**0.238**
	**Personality quality**	**13.609**	**25.032**	**0.000**	**0.221**
	**Intelligence**	**13.390**	**25.364**	**0.000**	**0.224**
Model financial status (M)	**Attractiveness**	**37.629**	**59.564**	**0.000**	**0.404**
	**Financial status**	**148.727**	**150.627**	**0.000**	**0.631**
	**Social status**	**128.880**	**140.151**	**0.000**	**0.614**
	**Personality quality**	**61.410**	**83.461**	**0.000**	**0.487**
	**Intelligence**	**106.383**	**122.400**	**0.000**	**0.582**
Observer’s financial status (O)	Attractiveness	5.866	0.912	0.406	0.020
	**Financial status**	**62.050**	**12.193**	**0.000**	**0.217**
	**Social status**	**47.416**	**9.173**	**0.000**	**0.173**
	**Personality quality**	**83.349**	**13.263**	**0.000**	**0.232**
	**Intelligence**	**63.868**	**10.632**	**0.000**	**0.195**
R × M	**Attractiveness**	**1.141**	**15.099**	**0.000**	**0.146**
	**Financial status**	**9.130**	**34.865**	**0.000**	**0.284**
	**Social status**	**10.217**	**38.176**	**0.000**	**0.303**
	**Personality quality**	**4.047**	**23.896**	**0.000**	**0.214**
	**Intelligence**	**7.596**	**29.573**	**0.000**	**0.252**
R × O	**Attractiveness**	**0.943**	**3.240**	**0.044**	**0.069**
	Financial status	1.257	2.234	0.113	0.048
	*Social status*	*1.622*	*2.890*	*0.061*	*0.062*
	Personality quality	1.086	1.997	0.142	0.043
	**Intelligence**	**1.632**	**3.092**	**0.050**	**0.066**
M × O	Attractiveness	0.852	1.349	0.265	0.030
	Financial status	0.891	0.902	0.409	0.020
	Social status	1.238	1.346	0.266	0.030
	*Personality quality*	*2.193*	*2.981*	*0.056*	*0.063*
	Intelligence	1.105	1.271	0.286	0.028
R × M × O	**Attractiveness**	**0.542**	**7.168**	**0.001**	**0.140**
	Financial status	0.226	0.863	0.426	0.019
	Social status	0.096	0.360	0.699	0.008
	Personality quality	0.129	0.759	0.471	0.017
	Intelligence	0.296	1.152	0.321	0.026

Note: Significant results are in bold; trending results are in italics.

**Table 6:** Mean (standard error) of facial attractiveness ratings and significance level of mate choice copying effect under each condition in Experiment 3.

Observer’s Financial Status	Low Financial Status Model			High Financial Status Model		
	Girlfriend	Classmate	*p*	Girlfriend	Classmate	*p*
Low	*3.469 (0.180)*	*3.364 (0.19)*	*0.098*	**3.968 (0.188)**	**3.784 (0.212)**	**0.028**
Medium	**3.052 (0.260)**	**2.588 (0.167)**	**0.011**	**3.868 (0.378)**	**3.344 (0.302)**	**<0.001**
High	3.042 (0.235)	3.026 (0.226)	0.848	**3.992 (0.302)**	**3.444 (0.258)**	**<0.001**

Note: Significant results are in bold; trending results are in italics.

**Table 7:** Mediating effect results of financial status, social status, personality, and intelligence under different financial status of observers.

Effect	Low Financial Status Observer				Medium Financial Status Observer				High Financial Status Observer			
	Effect	SE	Statistics	*p*	Effect	SE	Statistics	*p*	Effect	SE	Statistics	*p*
Financial status												
Total	**0.144**	**0.058**	***t* = 2.498**	**0.018**	**0.494**	**0.134**	***t* = 3.695**	**0.001**	**0.282**	**0.090**	***t* = 3.150**	**0.004**
Direct	0.045	0.048	*t* = 0.936	0.357	0.141	0.112	*t* = 1.262	0.218	0.029	0.046	*t* = 0.639	0.528
Indirect	**0.099**	**0.044**	***Z* = 2.267**	**0.023**	**0.353**	**0.116**	***Z* = 3.046**	**0.002**	**0.253**	**0.084**	***Z* = 3.014**	**0.003**
Social status												
Total	**0.144**	**0.058**	***t* = 2.498**	**0.018**	**0.494**	**0.134**	***t* = 3.695**	**0.001**	**0.282**	**0.090**	***t* = 3.150**	**0.004**
Direct	0.041	0.044	*t* = 0.928	0.361	0.148	0.100	*t* = 1.489	0.148	**0.076**	**0.034**	***t* = 2.221**	**0.035**
Indirect	**0.104**	**0.046**	***Z* = 2.256**	**0.024**	**0.336**	**0.117**	***Z* = 2.961**	**0.003**	**0.206**	**0.085**	***Z* = 3.046**	**0.016**
Personality												
Total	**0.144**	**0.058**	***t* = 2.498**	**0.018**	**0.494**	**0.134**	***t* = 3.695**	**0.001**	**0.282**	**0.090**	***t* = 3.150**	**0.004**
Direct	0.049	0.046	*t* = 1.056	0.300	0.145	0.110	*t* = 1.316	0.199	0.045	0.048	*t* = 0.933	0.359
Indirect	**0.095**	**0.044**	***Z* = 2.168**	**0.030**	**0.349**	**0.116**	***Z* = 3.019**	**0.003**	**0.237**	**0.083**	***Z* = 2.865**	**0.004**
Intelligence												
Total	**0.144**	**0.058**	***t* = 2.498**	**0.018**	**0.494**	**0.134**	***t* = 3.695**	**0.001**	**0.282**	**0.090**	***t* = 3.150**	**0.004**
Direct	*0.076*	*0.044*	*t = 1.754*	*0.090*	**0.188**	**0.060**	***t* = 3.148**	**0.002**	0.019	0.047	*t* = 0.409	0.686
Indirect	0.068	0.043	*Z* = 1.592	0.111	**0.228**	**0.050**	***Z* = 4.594**	**<0.001**	**0.263**	**0.084**	***Z* = 3.129**	**0.002**

Note: Significant results are in bold; trending results are in italics.

**Table 8:** Mediating effect results of financial status, social status, personality, and intelligence inference under different model financial status.

Effect	Low Financial Status Model				High Financial Status Model			
	Effect	SE	Statistics	*p*	Effect	SE	Statistics	*p*
Financial status								
Total	**0.194**	**0.068**	***t* = 2.845**	**0.006**	**0.416**	**0.063**	***t* = 6.605**	**<0.001**
Direct	**0.111**	**0.036**	***t* = 3.112**	**0.003**	**0.158**	**0.060**	***t* = 2.634**	**0.010**
Indirect	0.082	0.059	*Z* = 1.406	0.160	**0.258**	**0.052**	***Z* = 4.952**	**<0.001**
Social status								
Total	**0.194**	**0.068**	***t* = 2.845**	**0.006**	**0.416**	**0.063**	***t* = 6.605**	**<0.001**
Direct	**0.137**	**0.037**	***t* = 3.731**	**<0.001**	**0.154**	**0.059**	***t* = 2.591**	**0.011**
Indirect	0.057	0.058	*Z* = 0.989	0.323	**0.262**	**0.052**	***Z* = 5.005**	**<0.001**
Personality								
Total	**0.194**	**0.068**	***t* = 2.845**	**0.006**	**0.416**	**0.063**	***t* = 6.605**	**<0.001**
Direct	0.055	0.035	*t* = 1.569	0.120	**0.192**	**0.060**	***t* = 3.250**	**0.002**
Indirect	**0.139**	**0.060**	***Z* = 2.326**	**0.020**	**0.225**	**0.049**	***Z* = 4.552**	**<0.001**
Intelligence								
Total	**0.194**	**0.068**	***t* = 2.845**	**0.006**	**0.416**	**0.063**	***t* = 6.605**	**<0.001**
Direct	**0.120**	**0.037**	***t* = 3.273**	**0.002**	**0.188**	**0.060**	***t* = 3.148**	**0.002**
Indirect	0.074	0.058	*Z* = 1.274	0.203	**0.228**	**0.050**	***Z* = 4.594**	**<0.001**

Note: Significant results are in bold.

## Data Availability

The data that support the findings of this study are available on reasonable request from the corresponding author.
