# Correction: Zhou et al. (2025). Financial Status of Model, Target, and Observer Modulates Mate Choice Copying and the Mediating Effect of Personality. *Behavioral Sciences*, *15*(10), 1324

**DOI:** 10.3390/bs16010142

**Published:** 2026-01-20

**Authors:** Guomei Zhou, Shaxiao Ma, Di Wu

**Affiliations:** 1 Department of Psychology, Sun Yat-sen University, Guangzhou 510006, China; wudi68@mail3.sysu.edu.cn; 2 Guangzhou Nansha Qihui Special School, Guangzhou 511400, China; mashx3@mail3.sysu.edu.cn

## Missing Institutional Review Board Statement

In the original publication (Zhou et al., 2025), the Institutional Review Board statement was not included. It should state “The study was conducted in accordance with the Declaration of Helsinki and approved by the Ethical Review Committee for Protection of human Subjects of the Department of Psychology of Sun Yat-sen University (protocol code 2020-0325-0122, approved on 30 March 2020).”

## Missing Informed Consent Statement

In the original publication (Zhou et al., 2025), the Informed Consent was not included. It should state “Informed consent was obtained from all subjects involved in the study.”

The authors state that the scientific conclusions are unaffected. This correction was approved by the Academic Editor. The original publication has also been updated.
